# Impulse Control Disorders by Dopamine Partial Agonists: A Pharmacovigilance-Pharmacodynamic Assessment Through the FDA Adverse Event Reporting System

**DOI:** 10.1093/ijnp/pyac031

**Published:** 2022-05-27

**Authors:** Michele Fusaroli, Emanuel Raschi, Valentina Giunchi, Marco Menchetti, Roberto Rimondini Giorgini, Fabrizio De Ponti, Elisabetta Poluzzi

**Affiliations:** Pharmacology Unit, Department of Medical and Surgical Sciences, University of Bologna, Bologna, Italy; Pharmacology Unit, Department of Medical and Surgical Sciences, University of Bologna, Bologna, Italy; Pharmacology Unit, Department of Medical and Surgical Sciences, University of Bologna, Bologna, Italy; Pharmacology Unit, Department of Medical and Surgical Sciences, University of Bologna, Bologna, Italy; Department of Biomedical and Neuromotor Sciences, University of Bologna, Bologna, Italy; Pharmacology Unit, Department of Medical and Surgical Sciences, University of Bologna, Bologna, Italy; Pharmacology Unit, Department of Medical and Surgical Sciences, University of Bologna, Bologna, Italy; Pharmacology Unit, Department of Medical and Surgical Sciences, University of Bologna, Bologna, Italy

**Keywords:** Impulsive behavior, receptor, serotonin, 5-HT1A, aripiprazole, brexpiprazole, cariprazine

## Abstract

**Background:**

The dopaminergic partial agonism of the so-called third-generation antipsychotics (TGAs; aripiprazole, brexpiprazole, cariprazine) is hypothesized to cause impulse control disorders (ICDs). Relevant warnings by the Food and Drug Administration (FDA) were posted on aripiprazole (2016) and brexpiprazole (2018). Our study investigated the FDA Adverse Event Reporting System and the pharmacodynamic CHEMBL database to further characterize TGA-induced ICDs.

**Methods:**

We downloaded and pre-processed the FDA Adverse Event Reporting System up to December 2020. We adapted Bradford Hill criteria to assess each TGA’s —and secondarily other antipsychotics’—causal role in inducing ICDs (pathological gambling, compulsive shopping, hyperphagia, hypersexuality), accounting for literature and disproportionality. ICD clinical features were analyzed, and their pathogenesis was investigated using receptor affinities.

**Results:**

A total of 2708 reports of TGA-related ICDs were found, primarily recording aripiprazole (2545 reports, 94%) among the drugs, and gambling (2018 reports, 75%) among the events. Bradford-Hill criteria displayed evidence for a causal role of each TGA consistent across subpopulations and when correcting for biases. Significant disproportionalities also emerged for lurasidone with compulsive shopping, hyperphagia, and hypersexuality, and olanzapine and ziprasidone with hyperphagia. Time to onset varied between days and years, and positive dechallenge was observed in 20% of cases. Frequently, co-reported events were economic (50%), obsessive-compulsive (44%), and emotional conditions (34%). 5-Hydroxytryptamine receptor type 1a agonism emerged as an additional plausible pathogenetic mechanism.

**Conclusions:**

We detected an association between TGAs and ICDs and identified a new signal for lurasidone. ICD characteristics are behavior specific and may heavily impact on life. The role of 5-Hydroxytryptamine receptor type 1a agonism should be further explored.

## Introduction

### Third-Generation Antipsychotics (TGAs)

In the neuroscience-based nomenclature, aripiprazole, brexpiprazole, and cariprazine are classified together, and separately from other antipsychotics, because of their partial agonism on the dopamine receptors type 2 and 3 (D2/D3) and on the 5-hydroxytryptamine receptor type 1A (5-HT1A). Indeed, due to their unique pharmacodynamic profile, they are referred to as TGAs (Orsolini et al., 2020).

Aripiprazole is considered the prototype of TGAs. First approved for the treatment of schizophrenia (Food and Drug Administration [FDA], 2002; European Medicines Agency [EMA], 2004), it was subsequently extended to the treatment of manic episodes and relapse prevention in bipolar disorders. Cariprazine (FDA 2015; EMA 2017) shares the indications of aripiprazole, while brexpiprazole (FDA 2015; EMA 2018) is approved for schizophrenia and as an adjunctive treatment for major depressive disorder.

### TGA-Induced Impulse Control Disorders (ICDs)

While TGAs’ antagonist activity in high dopamine synaptic levels contributes to the therapeutic effect, their peculiar agonism in low levels accounts for a reduced risk of extrapyramidal syndrome (Aringhieri et al., 2018), which is a clinically important adverse event in D2-antagonist first-generation antipsychotics. Nonetheless, this partial agonism could be responsible for the onset of not well-defined behavioral addictions (ICDs), speculatively attributed to D3 agonism (Seeman, 2015) and already acknowledged as adverse reactions to dopamine agonists for Parkinson’s disease (Grall-Bronnec et al., 2018).

ICDs, defined as “the failure to resist an impulse, drive or temptation to perform an act that is harmful to the person or to others” (DSM-5), encompass a group of heterogeneous and not well-defined behaviors, including pathological gambling, hypersexuality, compulsive shopping, and binge eating, but also many other behavioral addictions and stereotypies that are inconstantly considered (Fusaroli et al., 2021). These conditions are often impulsive-compulsive degenerations of previous habits of the patients and can have a serious impact on their life, possibly resulting in family conflicts, divorce, loss of job and money, and legal problems. Therefore, patients should be informed about these side effects before beginning the therapy.

Evidence accumulated through the FDA Adverse Event Reporting System (FAERS) for aripiprazole-induced ICDs prompted the FDA to issue a relevant warning in 2016 (Moore et al., 2014; Etminan et al., 2017; Lertxundi et al., 2018; Center for Drug Evaluation and Research, 2019), followed by a label update for brexpiprazole in 2018, whereas cariprazine was not considered (Keks et al., 2020). The FAERS, which gathers spontaneous reports of adverse events from the entire world, is particularly suited to investigate rare or unexpected adverse events, such as ICDs, related to recently marketed drugs. It is also extensively used in the psychiatric area as a source of evidence, complementary to clinical trials, for safety profiling of antidepressants and antipsychotics (Poluzzi et al., 2013; Mazhar et al., 2019; Cepaityte et al., 2021; Gastaldon et al., 2021; Zazu et al., 2021) and for generating hypotheses on the underlying mechanisms (Aguiar et al., 2020; Gahr et al., 2021). Given that more than 5 years have passed from the first approval of brexpiprazole and cariprazine, the FAERS should provide enough data for a first in-depth assessment of TGA-related ICDs.

### Aim

The scope of this pharmacovigilance study is to extend current knowledge about TGA-induced ICDs, both as specific behaviors and as a unified diagnostic entity. Relying on FAERS spontaneous reports from real-world clinical practice and on CHEMBL pharmacodynamic measures, we aim to characterize ICDs’ clinical features and explore their underlying pharmacological basis. Our focus will be primarily on aripiprazole, brexpiprazole, and cariprazine, including a comparison with other antipsychotics.

## METHODS

### FDA Adverse Event Reporting System

The FAERS is a freely available spontaneous reporting system that collects worldwide reports of suspected adverse drug reactions. Its raw quarterly data (FDA, 2022) include demographic, therapeutic, and outcome details. Reactions and indications are coded using Medical Dictionary for Regulatory Activities (MedDRA) Preferred Terms.

All FAERS Quarterly Data (ASCII files from January 2004 up to December 2020) were downloaded and pre-processed for duplicate removal.

### Cases Retrieval

Because the MedDRA is redundant, the use of Standardized MedDRA Queries for case retrieval is advised. In the lack of Standardized MedDRA Queries for ICDs, we used MedDRA queries recently developed by combining a scoping review with pharmacosurveillance analyses (Fusaroli et al., 2021). We focused on the ICDs with the most robust clinical evidence: (1) pathological gambling (“gambling”, “gambling disorder”); (2) hypersexuality (“compulsive sexual behavior,” “sexually inappropriate behavior,” “hypersexuality,” “excessive masturbation,” “excessive sexual fantasies,” “libido increased,” “sexual activity increased,” “Kluver-Bucy syndrome”); (3) compulsive shopping (“compulsive shopping”); and (4) binge eating (“binge eating,” “food craving,” “hyperphagia”). We also extended the search to (5) a broader definition of ICDs, including routine and leisure activities (internet addiction, compulsive hoarding, walkabouts, excessive exercise, overwork), obsessive-compulsive and related disorders (stereotypy, body-focused repetitive behaviors, obsessive-compulsive disorders), conduct disorders (pyromania, kleptomania, aggressivity, paraphilia), drug abuse, and general terms concerning impulsivity and euphoria. Drugs of interest were aripiprazole, brexpiprazole, and cariprazine, but with an extended focus on all the antipsychotics included in the N05A category of the Anatomical Therapeutic Chemical classification updated in 2021.

Although we acknowledge that spontaneous reports do not allow to fully apply diagnostic criteria [in fact the need for diagnostic scales—for both ICDs and the individual behaviors affected—is still largely unanswered (Evans et al., 2019)], we presumed that any suspected adverse drug reaction, to be reported, must have some impact on the quality of life.

### Descriptive Analysis

To characterize TGA-related ICDs—both as a single heterogeneous diagnostic construct and as individual behaviors—we compared their demographics (sex, age, outcome, continent, reporter) with 2 Reference Groups (RGs): RG1, all other TGA reports (see Table 1); and RG2, all other ICD reports (see Table 2). Given that the population reporting TGA exposure was not equally split between the sexes and the tendency to report to the FDA is also skewed, even an equal tendency to report the adverse event would not result in equal distribution of cases between the sexes. Therefore, directly comparing the number of men with the number of women in the case group would not be informative. Women tend to report more often than men to the FAERS, and conditions for which a specific drug is prescribed may be distributed asymmetrically between men and women. To consider both reporting biases (e.g., higher reporting by women) and the sex distribution in the underlying indication for use, we chose as comparison the population of non-ICD TGA reports (RG1). In the same way, we compared TGA-related ICD reports with all the other reports of ICDs (RG2) to investigate whether ICDs related with TGAs share their features with other ICDs.

**Table 1.** TGA-induced ICDs, by Behavior^*a*^

Category	Pathological gambling n (%)	Hyper-sexuality n (%)	Compulsive shopping n (%)	Binge eating n (%)	TGA-related ICDs n (%)	Reference Group 1 n (%)
Cases	2018 (2.5)	920 (1.2)	1004 (1.3)	378 (0.5)	2708 (3.4)	76 796 (96.6)
Sex						
Female	902 (51.3)	390 (47.3)	557 (60.5)	229 (66.8)	1274 (53.2)	41 447 (60.0)
Male	855 (48.7)	435 (52.7)	363 (39.5)	114 (33.2)	1122 (46.8)	27 688 (40.0)
Missing	261 (–)	95 (–)	84 (–)	35 (–)	312 (–)	7661 (–)
*P* value	<.001	<.001	1.00	.08	<.001	
Continent						
North America	1896 (94.2)	776 (85.3)	980 (97.8)	302 (81.8)	2377 (88.6)	54 763 (74.3)
Europe	101 (5.0)	107 (11.8)	21 (2.1)	42 (11.4)	241 (9.0)	12 884 (17.5)
Asia	2 (0.1)	17 (1.9)	—	24 (6.5)	42 (1.6)	5139 (7.0)
Other	13 (0.6)	10 (0.9)	1 (0.1)	1 (0.3)	23 (0.8)	949 (1.3)
Missing	6 (–)	10 (–)	2 (–)	9 (–)	25 (–)	3061 (–)
*P* value	<.001	<.001	<.001	.033	<.001	
Reporter						
Consumer	656 (32.9)	246 (27.3)	174 (17.6)	218 (60.4)	1000 (37.7)	37 501 (52.0)
Medical doctor	119 (6.0)	94 (10.4)	44 (4.5)	53 (14.7)	250 (9.4)	16 456 (22.8)
Lawyer	1114 (55.8)	476 (52.8)	750 (76.0)	33 (9.1)	1162 (43.8)	708 (1.0)
Other^*b*^	106 (5.3)	85 (9.4)	19 (1.9)	57 (15.8)	242 (9.1)	17 421 (24.1)
Missing	23 (–)	19 (–)	17 (–)	17 (–)	54 (–)	4710 (–)
*P* value	<.001	<.001	<.001	<.001	<.001	
Age category						
<18 y	5 (0.8)	19 (5.6)	3 (1.2)	28 (12.4)	51 (4.7)	6329 (12.5)
Adult	613 (95.3)	301 (88.5)	243 (95.3)	188 (83.2)	975 (90.4)	38 747 (76.6)
Elderly	25 (3.9)	20 (5.9)	9 (3.5)	10 (4.4)	52 (4.8)	5514 (10.9)
Missing	1375 (–)	580 (–)	749 (–)	152 (–)	1630 (–)	26 206 (–)
*P* value	<.001	<.001	<.001)	.062	<.001	
Outcome						
Death or life-threat	33 (1.6)	19 (2.1)	16 (1.6)	18 (4.8)	57 (2.1)	6440 (8.4)
Disability	409 (20.3)	170 (18.5)	264 (26.3)	16 (4.2)	447 (16.5)	1743 (2.3)
Hospitalization	734 (36.5)	380 (41.3)	450 (44.8)	81 (21.4)	901 (33.3)	17 217 (22.4)
Other serious	426 (21.1)	201 (21.8)	162 (16.1)	95 (25.1)	614 (22.7)	20 181 (26.3)
Non-serious	416 (20.6)	150 (16.3)	112 (11.2)	168 (44.4)	689 (25.4)	31 215 (40.6)
*P* value	<.001	<.001	<.001	<.001	<.001	

Abbreviations: ICD, impulse control disorder; TGA, third-generation antipsychotic.

^*a*^Descriptive analysis of ICDs cases, separated by behavior, against other events reported for TGAs. The *P* value for the comparison of different behaviors against the reference group was calculated using the chi-square test, and a difference was deemed significant if *P* < .05 after the Holm-Bonferroni correction for multiple comparisons.

^*b*^Pharmacist, other healthcare professional, other non-specified.

**Table 2.** TGA-induced ICDs, Separated by TGA^*a*^

Category	Aripiprazole n (%)	Brexpiprazole n (%)	Cariprazine n (%)	TGA-related ICDS n (%)	Reference group 2 n (%)
Cases	2545 (21.6)	178 (1.5)	32 (0.3)	2708 (23.0)	9084 (77.6)
Sex					
Female	1172 (52.2)	117 (70.1)	21 (67.7)	1274 (53.2)	4773 (56.5)
Male	1073 (47.8)	50 (29.9)	10 (32.3)	1122 (46.8)	3676 (43.5)
Missing	300 (–)	11 (–)	1 (–)	312 (–)	635 (–)
*P* value	.004	.007	1.00	.042	
Continent					
North America	2219 (88.1)	177 (99.4)	28 (87.5)	2377 (88.6)	6264 (76.1)
Europe	237 (9.4)	—	4 (12.5)	241 (9.0)	1409 (17.1)
Asia	41 (1.6)	1 (0.6)	—	42 (1.6)	273 (3.3)
Other	23 (0.9)	—	—	23 (0.8)	289 (3.5)
Missing	25 (–)	0 (–)	0 (–)	25 (–)	849 (–)
*P* value	<.001	<.001	1.00	<.001	
Reporter					
Consumer	871 (35.0)	131 (73.6)	21 (65.6)	1000 (37.7)	5150 (61.8)
Medical doctor	232 (9.3)	21 (11.8)	7 (21.9)	250 (9.4)	1433 (17.2)
Lawyer	1162 (46.6)	7 (3.9)	2 (6.3)	1162 (43.8)	11 (1.3)
Other^*b*^	226 (9.1)	19 (10.7)	2 (6.3)	242 (9.1)	1641 (19.7)
Missing	54 (–)	0 (–)	0 (–)	54 (–)	749 (–)
*P* value	<.001	<.001	<.001	<.001	
Age category					
<18 y	50 (5.0)	1 (1.2)	—	51 (4.7)	280 (4.8)
Adult	910 (90.5)	73 (90.1)	14 (100.0)	975 (90.4)	4440 (76.3)
Elderly	45 (4.5)	7 (8.6)	—	52 (4.8)	1099 (18.9)
Missing	1540 (–)	97 (–)	18 (–)	1630 (–)	3265 (–)
*P* value	<.001	94	638	<.001	
Outcome					
Death or life-threat	54 (0.2)	4 (2.2)	—	57 (2.1)	317 (3.5)
Disability	446 (17.5)	3 (1.7)	1 (3.1)	447 (16.5)	310 (3.4)
Hospitalization	891 (35.1)	16 (9.0)	12 (37.5)	901 (33.3)	1380 (15.2)
Other serious	605 (23.8)	13 (7.3)	2 (6.2)	614 (22.7)	2808 (30.9)
Non-serious	549 (21.6)	142 (79.8)	17 (53.1)	689 (25.4)	4269 (47.0)
*P* value	<.001	<.001	.002	<.001	
Indication^*c*^					
Mood disorders	958 (77.6 vs 54.8)	108 (90.0 vs 84.6)	7 (58.4 vs 74.3)		
Psychotic disorders	277 (22.4 vs 45.2)	12 (10.0 vs 15.4)	5 (41.6 vs 25.7)		
Missing	1310 (–)	58 (–)	20 (–)	—	—
*P* value	<.001	.683	1.00		

Abbreviations: ICD, impulse control disorder; TGA, third-generation antipsychotic.

^*a*^Descriptive analysis of ICDs cases, separated by TGA, against ICDs events reported for other drugs. The *P* value for the comparison of different behaviors against the reference group was calculated using the chi-square test, and a difference was deemed significant if *P* < .05 after the Holm-Bonferroni correction for multiple comparisons.

^*b*^Pharmacist, other healthcare professional, other non-specified.

^*c*^The indication is compared with reports of the TGA without ICDs: n (% TGA-induced ICDs vs % TGA-induced other events).

Differences in categorical variables were assessed using a chi-squared test of independence performed on a 2 × 2 contingency table with Yates’ continuity correction (see supplementary Material—chi-squared statistics for the 2 × 2 tables comparing reports of men and women treated with TGAs with and without the ICD conditions). Significance was assumed when the *P* value, corrected for multiple comparisons using the Holm-Bonferroni method, was less than .05. To identify co-occurring conditions and the psychosocial impact of ICDs, co-reported psycho-social events were retrieved and discussed.

### Bradford Hill Causality Assessment

Adapted Bradford Hill criteria (Raschi et al., 2021), which account for already accrued evidence and disproportionality, were used to systematically gather evidence for TGA-induced ICDs (see Table 3 for a schematic presentation of these criteria and the strategies applied to assess them). Relying on the literature, we appraised biological plausibility and analogy to drugs already known for inducing ICDs. Through disproportionality analyses of the FAERS (reporting odds ratio [ROR]), we evaluated strength (extent of the ROR), consistency across sensitivity analyses (accounting for reporter, country, notoriety, and confounding by indication biases), coherence with the impulsivity substrate (“impulsive behavior,” “impulse-control disorder”), and speciﬁcity of the adverse event (assessing the association of ICDs with other antipsychotics). The ROR was calculated, using the 2 x 2 contingency table, whenever at least 3 reports with both the event and the drug investigated were found. A disproportionate reporting (i.e., an augmented probability of reporting an event with a specific drug) was considered significant when the lower limit of its 95% confidence interval was higher than 1. The ROR was reported as median (CI_2.5%_–CI_97.5%_) [n]. In particular, the analyses limited to reports submitted before the FDA warnings concerning ICD development with (1) aripiprazole (March 05, 2016); and (2) brexpiprazole (February 09, 2018), together with the analyses stratified by reporter occupation (i.e., medical doctor, consumer, lawyer), allowed us to partly adjust for the notoriety bias, likely amplified by the many related lawsuits. Furthermore, the possibility that TGAs are associated with ICDs not because they induce them but because of a common cause (i.e., their psychiatric indication for use) was considered through a subpopulation analysis limited to patients administered with antipsychotics. We also investigated temporal relationship (time to onset), biological gradient (association with daily dose, when available), reversibility of the adverse event (dechallenge and rechallenge), and the potential confounding role of drugs already acknowledged to cause ICDs (dopamine replacement therapy and previous treatment with other TGAs).

**Table 3.** Causality Assessment Procedure^*a*^

Criteria	Description	Method
Analogy	The drug belongs to a class known to give this adverse event, or it is similar to drugs known to induce it	Literature
Biological plausibility	The known molecular targets of the drug explain the pathogenesis of the event.	Literature
Empirical evidence	Empirical evidence in human or animal models.	Literature
Strength	The larger the association, the more likely that it is causal. Verified both on the entire database and considering only suspected drugs.	ROR
Consistency	Consistent findings are observed on different subpopulations taking the drug. The difference may be in reporter type (physician, patient, or lawyer) and country of occurrence (North America or elsewhere).	ROR
Coherence	Coherent findings are observed when investigating the association of the same drug with related events, in particular with an impulsivity substrate identified as “impulsive behavior” and “impulse-control disorder.”	ROR
Exclusion of bias^*b*^	The association between drug and event persists when correcting for notoriety bias (before the regulatory warnings on aripiprazole [before March 5, 2016], between the warning for aripiprazole and that for brexpiprazole [March 5, 2016–February 2, 2018], after the warning for brexpiprazole [after February 2, 2018]), and for channeling bias (only patients administered with antipsychotics).	ROR
Specificity	Among patients administered with antipsychotics, the association is specific for the drug considered and is not common to other antipsychotics.	ROR
Temporality	The event has to occur after the drug is administered, and the time to onset (delay from the first administration of the drug to the date of occurrence of the event) is coherent with biological and clinical notions.	Descriptive
Biological Gradient	There is a direct or inverse proportion between dose and occurrence of the event.	Descriptive
Reversibility	If the drug is stopped the event stops (dechallenge), and if the drug is reintroduced the event occurs again (rechallenge).	Descriptive
Exclusion of confounders^*b*^	The event is not always explained by the co-administration of dopamine replacement therapy or other drugs known to induce ICDs (aripiprazole and brexpiprazole).	Descriptive

Abbreviations: ICD, impulse control disorder; ROR, reporting odds ratio.

^*a*^Table showing the criteria adapted from Bradford Hill to assess causality in pharmacovigilance databases.

^*b*^Criteria not included in the original Bradford Hill’s Criteria.

To explore the pharmacological basis of ICDs, we integrated pharmacosurveillance and pharmacodynamic data. We interpolated intraclass RORs (limited to patients administered with antipsychotics) with Homo Sapiens affinity data (pKi) from the ChEMBL database (Mendez et al., 2019).

## RESULTS

### Descriptive Analysis

We found 2708 reports of TGA-related ICDs (23% of all the ICDs reported to the FAERS), each recording at least 1 ICD and at least 1 TGA (see Online Resource supplementary Figure 1 for the case-retrieval procedure). Among the adverse events reported (see Table 1), 2018 (74.5%) cases recorded pathological gambling, 1004 (37.1%) compulsive shopping, 920 (34.0%) hypersexuality, and 278 (10.3%) binge eating, with 1145 (42.3%) reports describing more than 1 ICD. Among suspected drugs (see Table 2), 2545 (94.0%) cases recorded aripiprazole (1564 depot), 178 (6.6%) brexpiprazole, and 32 (1.2%) cariprazine, with 45 (1.7%) reports describing more than 1 TGA. Using the broader definition, the cases increased to 5620: 1695 (30.2%) with conduct disorders (mostly aggressivity, 28 paraphilia, 6 kleptomania, 4 pyromania), 1489 (26.5%) with obsessive-compulsive and related disorders (mostly obsessive compulsive disorder, 164 trichotillomania, 131 dermatillomania), 785 (14.0%) with drug abuse, and 256 (4.6%) with leisure activities (241 compulsive hoarding, 15 poriomania).

Compared with RG1 (other TGA reports; Table 1), a significantly higher proportion of men was observed in TGA-related ICDs (*P *< .001, 47% vs 40%), particularly in gambling (*P* < .001, 49%) and hypersexuality (*P* < .001, 53%), with no significant difference in binge eating (*P* = .06, 33%) and compulsive shopping (*P* = 1, 40%). TGA-related ICDs were also significantly different in the geographical pattern (*P* < .001, 89% vs 74% North America) and the reporter type (*P* < .001, 44% vs 1% lawyers, 9% vs 23% physicians, 38% vs 52% consumers). Compulsive shopping reports were mostly reported by lawyers (76%). Binge eating reports were commonly submitted by consumers (60%). Finally, TGA-related ICDs also significantly differed from other TGA reports in age distribution and outcome (*P* < .001), with 90% adults (vs 77%), only 2% fatal outcomes (vs 8%), and more common disability and hospitalization (17% vs 2%, and 33% vs 22%, respectively), particularly in compulsive shopping (26% and 45%).

Compared with RG2 (other ICD reports; Table 2), TGA-related ICDs had a significantly higher proportion of men (*P* = .033, 47% vs 40%), except for brexpiprazole (*P* = .007, 70% women). Significant differences were also observed for geographical distribution (*P* < .001, 89% vs 76% from North America), age distribution (*P* < .001, 90% vs 76% adults), and reporter type (*P* < .001, 44% vs 1% lawyers, 9% vs 17% physicians, 38% vs 62% consumers). Consumers’ contribution was higher for brexpiprazole (*P* < .001, 74%) and cariprazine (*P* < .001, 66%). Finally, disability and hospitalization in TGA-related ICDs were higher than for other ICDs (*P* < .001, 17% vs 3%, and 33% vs 15%). Concerning the indication for use, mood disorders were significantly more represented in aripiprazole-related ICDs (*P* < .001, 78% vs 55% in other suspected reactions to aripiprazole). No significant difference was observed, however, for brexpiprazole (*P* = .68, 90% vs 85%) and cariprazine (*P* = 1, 58% vs 74%). The temporal trend analysis showed a peak of submissions by consumers in 2017 and by lawyers in 2018 (see supplementary Figure 2 for additional information regarding temporal trends). The more frequently co-reported events concerned economic (50%), obsessive-compulsive (44%), emotional conditions (34%), unspecified mental disorders (30%), and suicidal and self-injurious behaviors (29%). See supplementary Table 1 for additional information on co-occurring events.

### Bradford Hill Criteria

Strong disproportionality was found for ICDs (strength) and for general impulsivity (coherence) with each TGA and was consistent across subpopulations (consistency). The RORs were higher for aripiprazole, followed by brexpiprazole and cariprazine (see Table 4). Even if RORs increased when calculated from lawyers’ reports and after FDA warnings, the significance was preserved when restricting to reports submitted before the warnings and by physicians. The intraclass RORs (disproportionality limited to patients using N05A drugs) showed a significant association between TGAs and ICDs, both as a unitary diagnostic entity and as individual behaviors (correction for bias). Other antipsychotics associated with the ICD unitary construct (specificity) were lurasidone (121 cases; associated with hyperphagia, hypersexuality, and compulsive shopping), olanzapine and ziprasidone (associated with hypherphagia), and iloperidone (associated with pathological gambling). Refer to supplementary Figure 3 for the distribution of antipsychotic-related ICDs among different behaviors, and supplementary Figures 4 and 5 for antipsychotics intraclass disproportionality. Possible confounders were co-reported in 55 reports of aripiprazole (2.2%, mostly ropinirole and pramipexole). Thirty-six reports of brexpiprazole (20.2%) recorded an exposition to aripiprazole. Of the 32 reports of cariprazine, 9 also recorded aripiprazole or brexpiprazole, and 2 with both (exclusion of confounders). Dechallenge (ICD resolution after TGA discontinuation) occurred in more than 20% cases, reaching 40.6% for cariprazine (reversibility), whereas a positive rechallenge (ICD reappearance after TGA reintroduction) was recorded in only 1%–3% of the reports. The delay from the first administration of the TGA and the onset of the ICD ranged between days and years. The median time to onset was 89, 8, and 15 days for aripiprazole, brexpiprazole, and cariprazine, respectively (temporality), with median daily doses of 10, 1, and 4.5 mg (other suspected reactions to cariprazine developed within an interquartile dose range of 1.5–3 mg; biological gradient). Literature evidence consisted of analogy to dopamine agonists; empirical, mostly observational, evidence; and biological plausibility related to the catecholaminergic activity. In conclusion, aripiprazole and brexpiprazole satisfied Bradford Hill criteria for causality assessment, apart from biological gradient (brexpiprazole had also little empirical evidence). Cariprazine, instead, satisfied all the criteria apart from empirical evidence. An extract of the 32 cariprazine cases is shown in the supplementary Table 2.

**Table 4.** Causality Assessment Results^*a*^

Criteria	Aripiprazole	Brexpiprazole	Cariprazine^*b*^
Analogy	Cariprazine belongs to TGAs together with aripiprazole and brexpiprazole, already known for a plausible role in inducing ICDs. Furthermore, their pharmacodynamic profile partially overlaps with that of dopamine agonists, known to induce ICDs.		
Biological plausibility	TGAs, unlike other antipsychotics, are partial agonists of D2 and D3 receptors and have a broader action on other catecholaminergic receptors. Thus, they do interact with systems thought to be the neuroanatomical correlates of impulsivity.		
Empirical evidence	Aripiprazole case reports (Mahapatra et al., 2016), pharmacosurveillance on FAERS and EudraVigilance (Moore et al., 2014; Lertxundi et al., 2018) and FDA warning (Center for Drug Evaluation and Research, 2019), epidemiological studies (Etminan et al., 2017)	Brexpiprazole pharmacosurveillance on EudraVigilance (Zazu et al., 2021) and FDA warning	Cariprazine pharmacosurveillance on EudraVigilance (Zazu et al., 2021)
Strength			
All	41.3 (39.5–43.2) [2545]	24.0 (20.7–27.9) [178]	11.6 (8.2–16.5) [32]
Suspected	101.9 (97.4–106.6) [2449]	45.3 (38.9–52.8) [170]	21.4 (14.5–31.6) [26]
Consistency			
Physician	25.5 (22.2–29.3) [232]	25.1 (16.2–38.7) [21]	29.9 (14.1–63.3) [7]
Consumer	23.5 (21.9–25.3) [871]	23.0 (19.3–27.4) [131]	10.4 (6.7–16.0) [21]
Lawyer	2043.6 (1659.2–2517.1) [1162]	95.1 (34.4–262.5) [7]	[2]
US	54.6 (52.0–57.3) [2219]	25.0 (21.5–29.1) [177]	10.7 (7.3–15.5) [28]
Elsewhere	15.3 (13.6–17.2) [326]	[1]	26.2 (9.7–70.9) [4]
Coherence	23.0 (20.9–25.2) [522]	8.0 (5.2–12.5) [20]	7.7 (3.7–16.3) [7]
Exclusion of bias			
Before 2016	12.5 (10.7–14.6) [168]	17.2 (8.1–36.2) [7]	[0]
2016-2018	69.2 (63.3–75.6) [806]	18.6 (14.3–24.2) [58]	9.4 (3.9–22.8) [5]
After 2018	136.3 (127.0–146.3) [1433]	33.4 (27.6–40.4) [113]	13.4 (9.2–19.7) [27]
Intra-N05A	16.6 (15.6–17.8) [2545]	4.9 (4.2–5.7) [178]	2.3 (1.6–3.3) [32]
Specificity^*c*^	Even if on the whole FAERS disproportionality can be found for many antipsychotics, among patients administered with antipsychotics, only a few remain significant: the 3 TGAs and lurasidone, and with a more ambiguous signal olanzapine and ziprasidone (and iloperidone with a broader interval and the lower limit near to 1).		
Temporality	89 (8-524) d [188]	8 (2-22) d [12]	15 (2-55) d [7]
Biological gradient	10 (5-15) mg [1265] vs 10 (5-15) mg [10 988]	1 (0.875-2) mg [80] vs 1 (0.5-2) mg [2752]	4.5 (3-6) mg [11] vs 1.5 (1.5-3) mg [667]
Reversibility			
Dechallenge	601 (23.6%)	44 (24.7%)	13 (40.6%)
Rechallenge	76 (3.0%)	2 (1.1%)	1 (3.1%)
Exclusion of confounders	55 (2.2%) Dopamine replacement therapy [55]	36 (20.2%) Aripiprazole [36]	11 (34.4%) Aripiprazole [10] Brexpiprazole [3]

Abbreviations: FAERS, FDA Adverse Event Reporting System; FDA, US Food and Drug Administration; ICD, impulse control disorder; ROR, reporting odds ratio; TGA, third-generation antipsychotic.

^*a*^Using the procedure described in Table 3, a causality assessment was performed for the 3 dopamine partial agonists. Reporting odds ratios were reported as median (CI_2.5%_–CI_97.5%_) [n]. Time to onset and biological gradient were reported as median (Q_1_–Q_3_) [n]. Reversibility and confounders were reported as n (%) on the number of cases. The occurrences of the main confounders were also reported.

^*b*^See supplementary Figure 3 for individual cariprazine cases.

^*c*^See supplementary Figures 4 and 5 for significant RORs of other antipsychotics.

**Figure 1.**
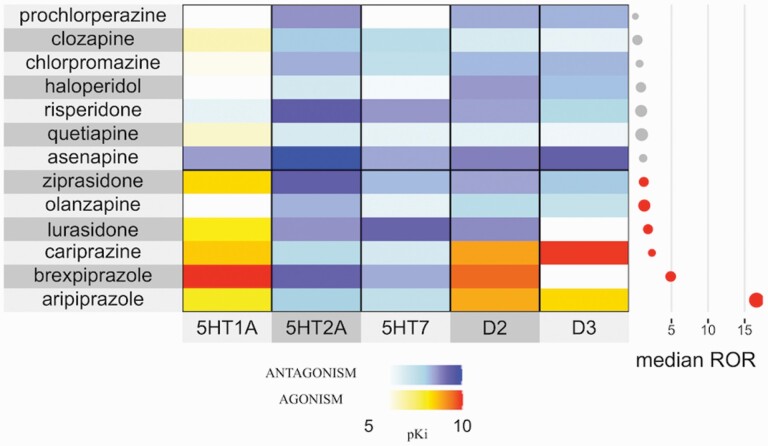
Potential mechanisms underlying impulse control disorders (ICDs). Heat map comparing disproportionalities and receptor activity of multiple antipsychotics. Agonism and partial agonism are shown in warm tones, antagonism in cold tones, with intensity proportional to the affinity (pKi). The adjacent plot shows significant (in red) and non-significant (in grey) disproportionalities.

The interpolation of disproportionalities and affinity towards different receptors (Figure 1) highlighted a common agonism for dopaminergic receptors (especially D2) shared by TGAs. The only molecular target shared also with lurasidone and other ICD-associated antipsychotics was 5-HT1A.

## Discussion

### Further Evidence for TGA-Induced ICDs

To gather further evidence on the potential association between TGAs and ICDs, we adapted Bradford Hill criteria into a 12-point scheme for evidence assessment by combining literature and pharmacosurveillance disproportionality analyses.

The literature had already accrued strong observational evidence supporting the plausibility of a causal link between aripiprazole and ICDs (Moore et al., 2014; Mahapatra et al., 2016; Etminan et al., 2017; Lertxundi et al., 2018), whereas brexpiprazole and cariprazine were supported by only weak pharmacosurveillance evidence [7 cases each and significant RORs on the EudraVigilance database (Zazu et al., 2021)]. Nonetheless, all the TGAs partially share their pharmacodynamic profile with dopamine agonists, known to induce ICDs; partial agonism on D2/D3 receptors, along with a variegate catecholaminergic activity, could be responsible for influencing the neuroanatomical correlates of impulsivity.

Our study contributes with stronger, multifaceted, pharmacosurveillance evidence. TGAs were recorded in one-quarter of the ICDs in the FAERS. A total 94% of the cases concerned patients exposed to aripiprazole. This drug has a long marketing life, a well-established notoriety, and is more commonly administered due to guideline promotion. Interestingly, the FDA warning concerning aripiprazole resulted in a strengthening of the ROR limited to aripiprazole. After the warning concerning brexpiprazole, a strengthening of the ROR was also observed for the other TGAs (known as ripple effect), maybe because it highlighted the plausibility of a drug class-related effect. Time to onset for cariprazine and brexpiprazole was coherent with what is known of aripiprazole (days to several months) (Grall-Bronnec et al., 2016), and a high dechallenge rate was documented. The co-recording of multiple TGAs in 1.7% of the reports, plausibly referring to antipsychotic switching, complicated the assessment of newer TGAs. Doses were coherent with approved posology, and cariprazine cases reported higher doses than non-cases. Disproportionalities were significant for all TGAs, satisfying strength, consistency, coherence, and exclusion of bias criteria. The lower but significant intraclass ROR supported the existence of a disease-related susceptibility to ICDs not sufficient to explain the disproportionality of TGAs, lurasidone [a recent antipsychotic for which a case of hypersexuality has already been recorded (Reddy et al., 2018)], and olanzapine and ziprasidone (limited to hyperphagia). The high proportion of aripiprazole-related ICD cases in which the primary indication for use was a mood disorder is in line with the hypothesis that depression may be a synergistic risk factor for impulsive-compulsive behaviors (Cao et al., 2021).

Notably, TGAs and lurasidone are often considered safe alternatives to SGAs in patients fearing a bodyweight increase. In fact, both aripiprazole and lurasidone are classified in the antipsychotics group with the lowest risk for weight increase (Musil et al., 2015). Nonetheless, the occasional development of TGA-induced hyperphagia may be sufficient to determine weight gain, even in the lack of metabolic dysfunction. It may therefore be indicated to maintain careful weight monitoring in the early phases after the switch. Furthermore, a strict monitoring should be extended to all the drugs with a pharmacodynamic profile overlapping with that of TGAs. For example, lumateperone is a new multimodal agent characterized by dopaminergic presynaptic partial agonism and postsynaptic antagonism (Snyder et al., 2015). In part because of its recent approval (FDA, 2019), we found only 151 reports concerning lumateperone, with no record of ICDs.

### Clinical Feature of TGA-Induced ICDs

A total of 42.3% of the TGA-related ICD reports recorded more than 1 ICD. This proportion is higher than that observed in dopamine-agonist–related ICDs in Parkinson’s disease (approximately 25%) (Weintraub et al., 2010) and may be explained both by differences in the underlying population and by under-reporting of individual, less-impactful ICDs.

The influence of culture on the insurgence and clinical manifestation of ICDs is strong and already acknowledged (El Otmani et al., 2019). In agreement with past evidence, the high contribution of reports from North America, particularly in compulsive shopping and pathological gambling, could be related to a strong consumeristic culture (Parra-Díaz et al., 2020). FAERS data were also consistent with expected gender patterns (Weintraub et al., 2010; Santangelo et al., 2013), with men significantly more likely to experience hypersexuality and pathological gambling relative to other TGA reports. Instead, we failed to find a significantly higher proportion of binge eating and compulsive shopping in females. Gender patterns may be in part explained by gender norms, intended as “social norms defining acceptable and appropriate actions for women and men in a given group or society” (Cislaghi and Heise, 2020), and may therefore change across countries and time. Although ICDs are rarely related to fatal outcomes, disability and hospitalization are reported far more frequently than in other TGAs adverse events. Disability may reflect the strong impact that ICDs have on the life and functioning of patients and their caregivers and can be also observed in the pattern of co-reported events: “economic circumstances,” “anxiety,” “employment issues,” “disability,” “family and partner issues,” “housing circumstances,” and “criminal activity.” The fact that binge eating results in less disability raises the question as to whether weaker stigma could reduce the disability related to other ICDs. The higher proportion of hospitalization may instead be related with the fact that 29% of the ICD cases (vs 5% non-cases; data not shown) recorded also self-injurious behaviors, with 734 reports recording “suicidal ideation,” 670 “suicide attempt,” and only 6 “suicide completed” (which explains the low proportion of deaths).

The strong contribution of reports by lawyers, instead, does not seem to be related only to the seriousness of ICDs, because ICDs induced by other drugs (e.g., dopamine agonists) have a low contribution by lawyers. Instead, this could be the result of both the over-signaling of lawyers, using the ICD diagnosis to defend their clients, and the sub-signaling of physicians, who consider ICDs as part of the illness and not a drug reaction. Hopefully the increase in the rate of reports by doctors and consumers regarding brexpiprazole and cariprazine, compared with aripiprazole, may imply an increasing awareness.

Finally, other conditions frequently co-reported support the use of an extended query for ICDs cases (eating disorders; ICDs; conduct disorders; obsessive-compulsive symptoms, such as hoarding, trichotillomania, dermatillomania; and suicidal and self-injurious behaviors). Therefore, we call for an effort to better define ICDs toward more standardized and sensitive studies.

### Insights in ICD Pathogenesis

Of note, lurasidone, along with brexpiprazole, does not share with aripiprazole and cariprazine the partial agonism on D3 receptors, which is strongly advocated as the primary mechanism in the onset of dopaminergic agent–induced ICDs (Seeman, 2015). Instead, it shares a strong agonism on 5-HT1A (Huang et al., 2012), an inhibitory auto-receptor located in the dorsal raphe. 5-HT1A inhibits the serotonergic circuit, which is usually responsible for the inhibition of the dopaminergic neurons departing from the ventro-tegmental area and directed to the ventral striatum. Therefore, 5-HT1A may inhibit the pathway responsible for impulse control. This is coherent with the finding, in Parkinson’s disease, that ICDs are associated with an increased release of dopamine in the ventral striatum (Martini et al., 2018), which opposes the prevailing hypothesis of a primary role related to the exogenous dopaminergic activity. Interestingly, different studies have already demonstrated 5-HT1A–induced impulsivity and addiction in mice (Humby et al., 2020; Chu et al., 2021) and investigated the relationship between impulsivity and 5-HT1A polymorphisms in humans (Benko et al., 2010; Stamatis et al., 2020). Furthermore, 5-HT1A agonists such as buspirone and vortioxetine may cause impulsive-compulsive behaviors in rats (Liu et al., 2004; Lu et al., 2013; Martis et al., 2021), and a 5-HT1A partial agonist (tandospirone) may result in reduced impulsivity in rats because of its 5-HT1A antagonistic activity (Ohmura et al., 2013). Therefore, based on our pharmacovigilance analyses and on the supporting literature, we raise the hypothesis of 5HT1A agonism as a plausibly pivotal mechanism of the disinhibition proper of TGA-related ICDs, and 5-HT1A antagonism as a promising approach for therapeutic intervention. Nonetheless, we acknowledge that a high pKi does not always result in high receptor occupancy and that positron emission tomography occupancy studies found undetectable occupancy for brexpiprazole on both D3 and 5-HT1A receptors (Girgis et al., 2020). Therefore, further studies investigating the pharmacodynamics of TGAs and other drugs involved in the development of ICDs are required.

### Strengths and Limitations

The analysis of spontaneous adverse events reports, peculiar to pharmacosurveillance studies, does not allow formal risk assessment and requires considering multiple biases. In particular, researchers must contend with unverified reports, inter-reporter heterogeneity in the choice of terms, under-reporting, and the influence of mass media and regulatory warnings (i.e., notoriety bias). Nonetheless, it allows to collect great numbers of cases from a large heterogeneity of environments and to account for clinical errors, populations under-represented in clinical trials, comorbidity, and interactions. Through our in-depth analysis and adapted Bradford Hills Criteria, we integrated multiple data from the FAERS database to provide more evidence regarding TGA-induced ICDs. Although acknowledging the heterogeneous compilation of the reports, we investigated the potential role of the drugs in determining the event without neglecting the predisposition of the individual, related to concomitants and underlying disease. Note that other kinds of predisposition, such as genetic or environmental, could not be assessed through the limited information of the reports. As Hill himself clearly understood, the validation of these criteria supports evidence of a causal relationship, but no amount of evidence will ever be enough to confirm a suspicion, nor will a lack of evidence will ever be enough to dismiss it. Hence, further clinical studies are required.

### Conclusion

This study gathered FAERS data and literature evidence to assess the link between TGAs and ICDs and raised several insights and hypotheses deserving further investigation. Our findings, even if not sufficient to establish a causality, are adjusted for major biases and can help to better define the path for regulatory actions towards a more conscious and safer use of TGAs. The fulfilled Bradford Hill criteria support the warning already issued and highlight, for the first time—to our knowledge—the plausible role of cariprazine in inducing ICDs. In particular, when switching to TGAs because of SGA-induced weight gain, early detection is important of any potential surge in time spent eating, which could itself be responsible for weight gain. Lurasidone also displays associations with individual and grouped ICDs.

A better ICDs syndrome characterization is pivotal to support monitoring of the patient by the caregivers. The features of ICD reports are consistent with what is already known from clinical studies, with sex differences in the ICD manifestation. The analysis of co-reported events supports the strong impact of ICDs on the social and psychological life of individuals and their families and points to the need for an extended Standardized MedDRA Query able to detect more ICD reports.

Finally, a promising target to understand and treat ICDs is also represented by 5-HT1A, which may be responsible for the disinhibition, resulting in the progression from an everyday gratifying behavior toward an ICD and, eventually, a compulsion. Further knowledge about ICD mechanisms may be extremely valuable to a more conscious switch between antipsychotics and to the search of pharmacological treatments for ICDs among new and repurposed drugs.

## Supplementary Material

pyac031_suppl_Supplementary_Material

## Data Availability

The pharmacovigilance data are freely accessible on the FDA website: https://fis.fda.gov/extensions/FPD-QDE-FAERS/FPD-QDE-FAERS.html. The code used is made available on request to the author.
