# Supplementary material for: Impulse Control Disorders by Dopamine Partial Agonists: A Pharmacovigilance-Pharmacodynamic Assessment Through the FDA Adverse Event Reporting System
Source: Int J Neuropsychopharmacol. 2022 May 27;25(9):727–36. doi: 10.1093/ijnp/pyac031 (PMC9515127; doi:10.1093/ijnp/pyac031)
Supplement: pyac031_suppl_Supplementary_Material [file pyac031_suppl_supplementary_material.pdf]

Figure S1. Flowchart showing the procedure for case-retrieval.

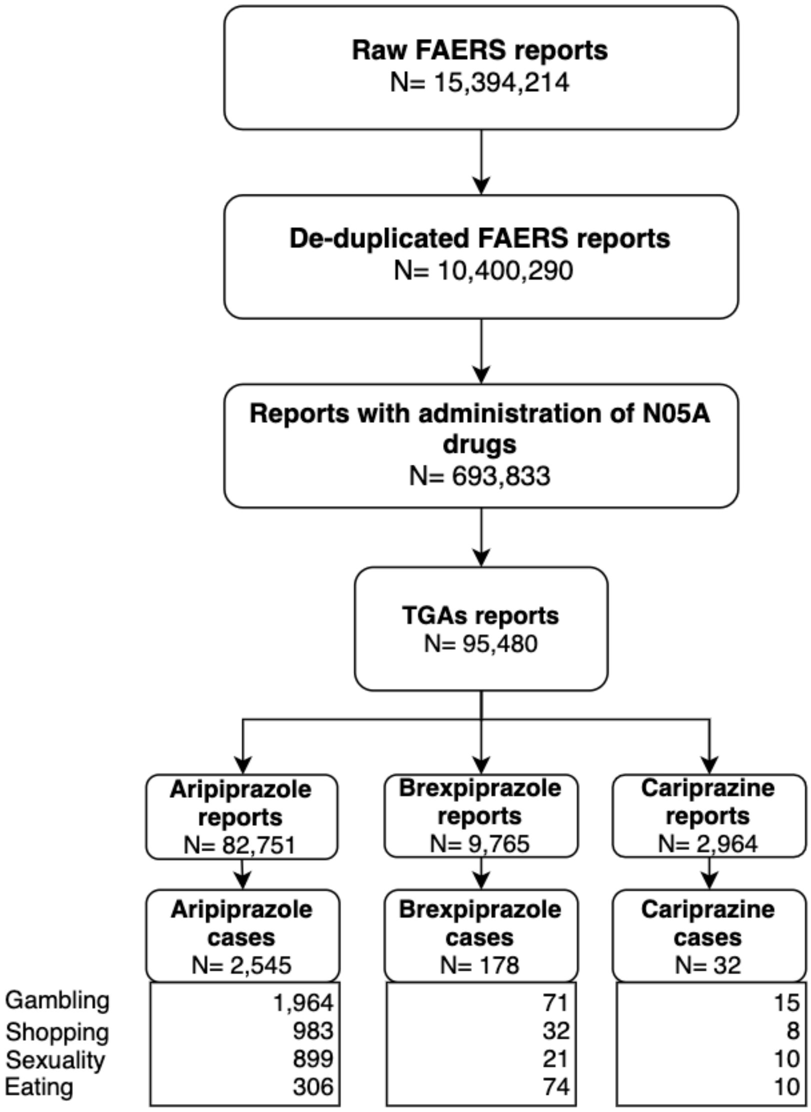

Figure S2. TGAs-induced ICDs reports through time, segregated by reporter type.
CN = patient, LW = lawyer, MD = physician, OT = other

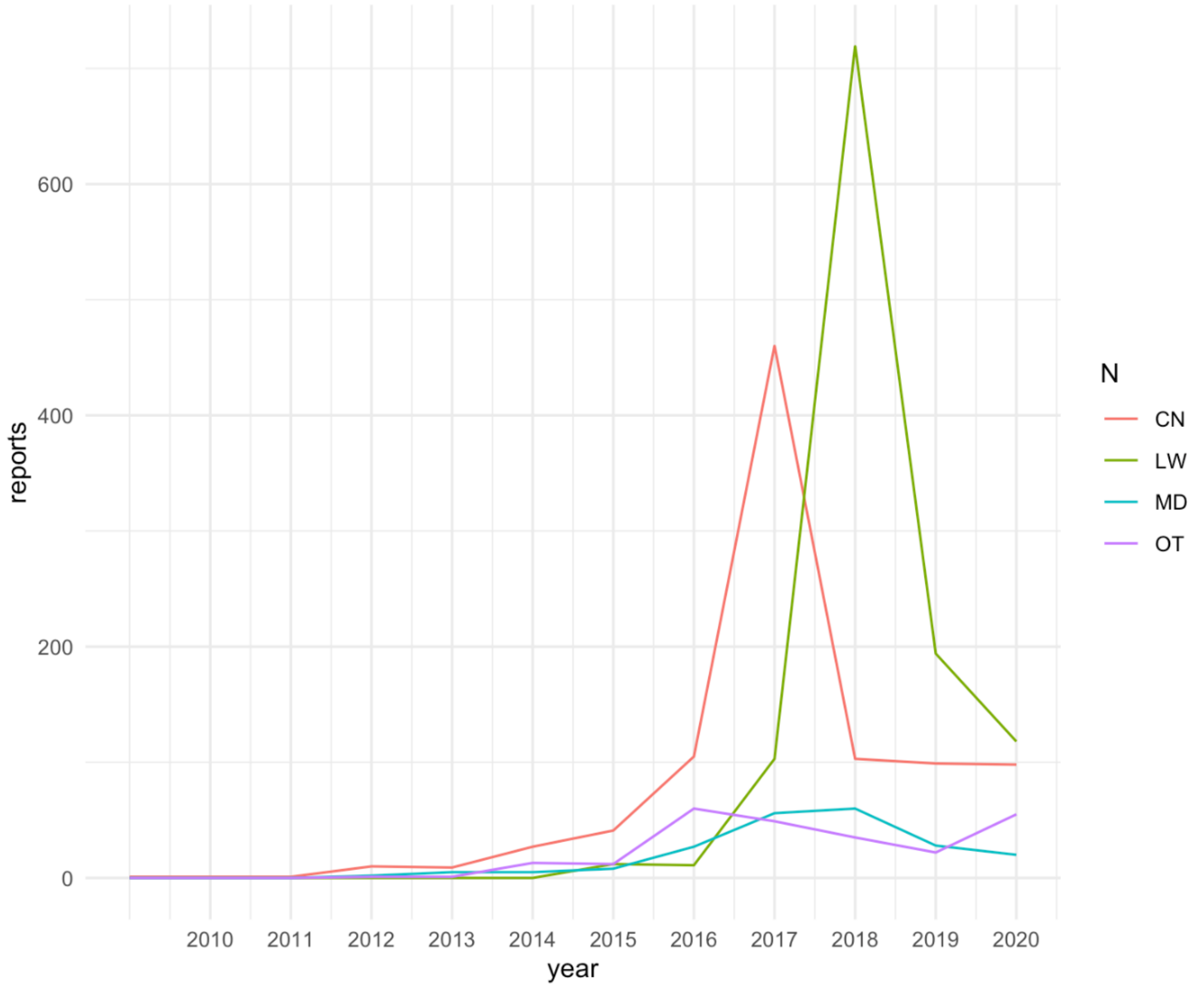

Table S1. Other events co-occurring with ICDs, segregated by High-Level Term.
% has been calculated on the number of TGAs-related ICDs reports

HLT	%	PTs
economic circumstances	50	economic problem (1331);bankruptcy (375);inability to afford medication (13);insurance issue (7);poverty (1)
obsessive-compulsive disorders and symptoms	44	obsessive-compulsive disorder (1089);compulsive hoarding (240);trichotillomania (152);dermatillomania (125);compulsions (18);obsessive thoughts (12);body dysmorphic disorder (1);obsessive-compulsive symptom (1)
emotional and mood disturbances nec	34	emotional distress (798);irritability (88);anger (37);mood altered (18);euphoric mood (16);emotional disorder (15);frustration tolerance decreased (11);emotional poverty (7);dysphoria (6)
mental disorders nec	30	mental disorder (822);mental status changes (1)
suicidal and self-injurious behaviour	29	suicidal ideation (734);suicide attempt (670);intentional self-injury (15);self-injurious ideation (9);completed suicide (6);suicidal behaviour (3)
anxiety symptoms	26	anxiety (641);stress (55);agitation (52);nervousness (11);tension (1)
eating disorders nec	21	eating disorder (571);merycism (3);bulimia nervosa (2)
employment issues	17	loss of employment (442);unemployment (5);job dissatisfaction (3);stress at work (2);threat of redundancy (1)
mood alterations with depressive symptoms	17	anhedonia (382);depressed mood (56);feeling guilty (14);tearfulness (13);decreased interest (8);depressive symptom (6);feelings of worthlessness (4);feeling of despair (2);negative thoughts (1);psychomotor retardation (1)
disability issues	16	disability (407);physical disability (30);loss of personal independence in daily activities (22);impaired work ability (13);impaired driving ability (1);hearing aid user (1);wheelchair user (1);mental disability (1)
impulse control disorders	12	impulsive behaviour (199);impulse-control disorder (153);intermittent explosive disorder (1)
family and partner issues	11	divorced (267);marital problem (16);partner stress (15);family stress (6);pregnancy of partner (1)
housing circumstances	9	homeless (242)
criminal activity	9	theft (201);shoplifting (178);legal problem (24);imprisonment (21);sexual abuse (6);crime (5);physical assault (3);drug diversion (2)
disturbances in initiating and maintaining sleep	7	insomnia (166);middle insomnia (12);initial insomnia (3);hyposomnia (1)
social issues nec	7	personal relationship issue (103);social problem (94);impaired quality of life (14);refusal of treatment by patient (8);poor personal hygiene (7);substance use (6);exercise lack of (2);prostitute (2);patient uncooperative (1)
mental disorders due to a general medical condition nec	6	neuropsychiatric symptoms (161);neuropsychiatric syndrome (3)
depressive disorders	6	depression (153);major depression (19);persistent depressive disorder (9);perinatal depression (1)
behaviour and socialisation disturbances	5	aggression (64);paranoia (34);disinhibition (13);hostility (13);social avoidant behaviour (8);homicidal ideation (8);antisocial behaviour (6);grandiosity (6);personality change (5);soliloquy (3);violence-related symptom (3);negativism (2);suspiciousness (2);disturbance in social behaviour (1);indifference (1);impatience (1)
substance related and addictive disorders	5	alcoholism (32);drug dependence (31);dependence (28);alcohol abuse (25);drug abuse (23);substance abuse (15);substance dependence (9);nicotine dependence (7);behavioural addiction (4);tobacco abuse (3);drug use disorder (2);alcohol use disorder (2)

Figure S3. Distribution of individual maladaptive behaviors in antipsychotic associated ICDs.

Rate of ICDs on the number of reports on the left, behavioral distribution on the right. Only the first 10 antipsychotics in order of case number, plus cariprazine, were shown.

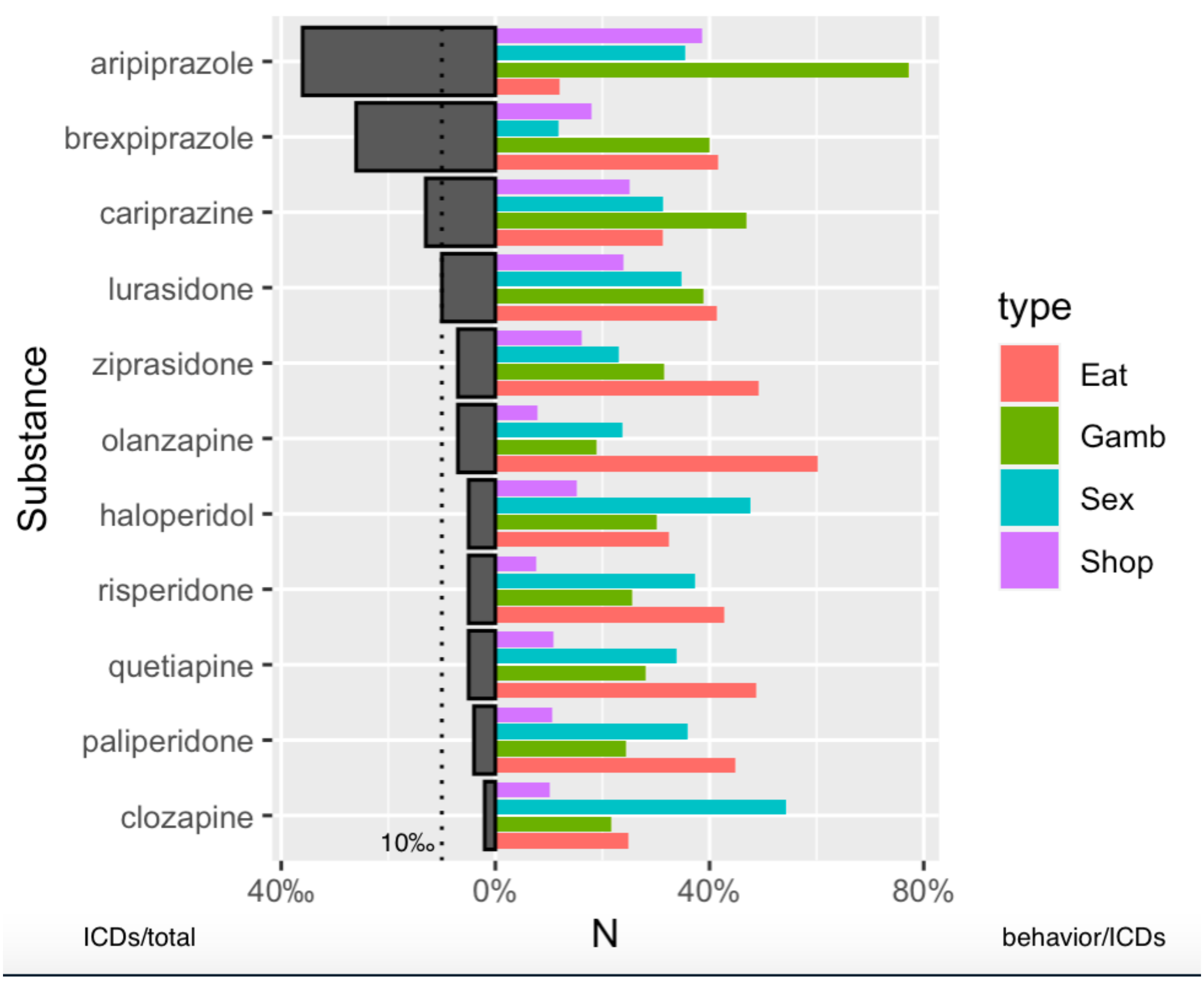

Figure S4. Antipsychotics associated with ICDs

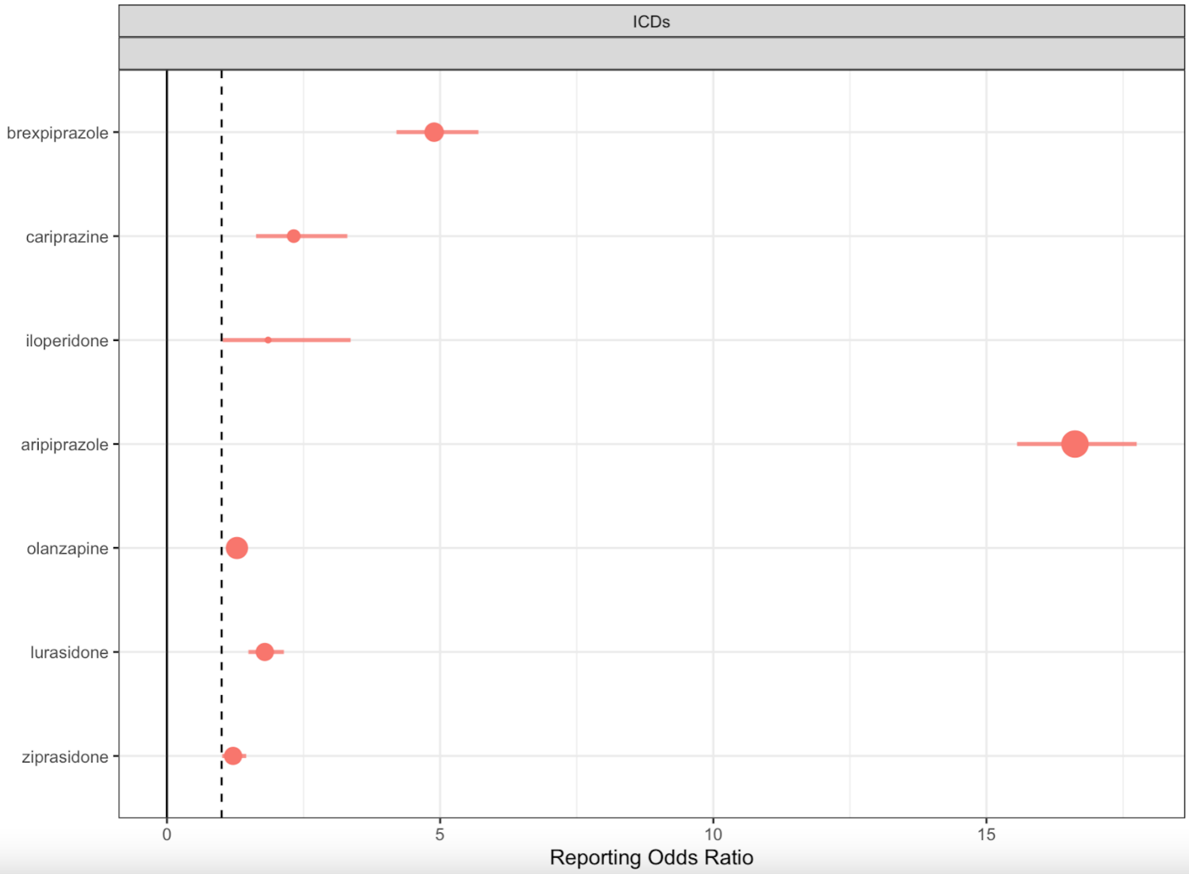

Substance	ROR
ziprasidone	1.21 (1.01-1.45) [118]
lurasidone	1.79 (1.49-2.14) [121]
olanzapine	1.28 (1.16-1.41) [442]
aripiprazole	16.62 (15.56-17.75) [2545]
iloperidone	1.85 (1.02-3.36) [11]
cariprazine	2.32 (1.63-3.3) [32]
brexpiprazole	4.89 (4.2-5.7) [178]

Figure S5. Antipsychotics associated with individual maladaptive behaviors

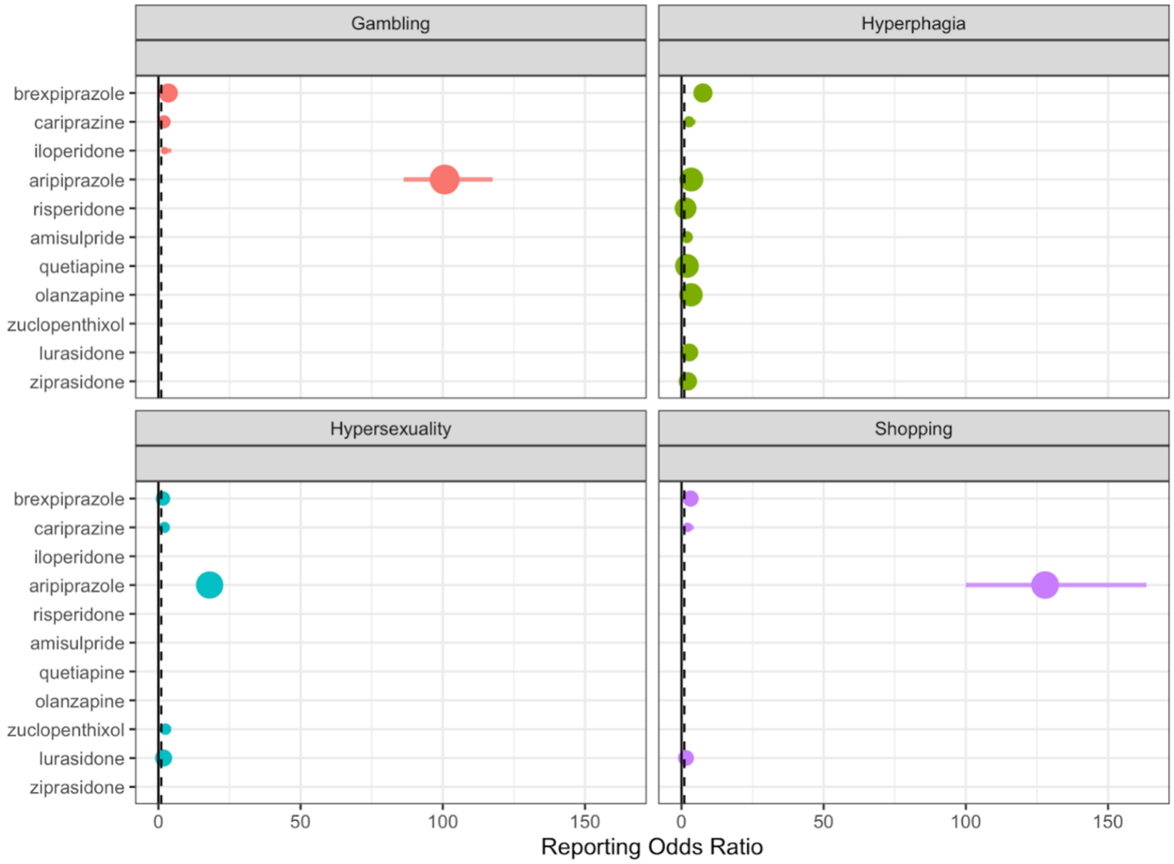

Shopping	
**Substance**	**ROR**
lurasidone	1.59 (1.1-2.3) [29]
aripiprazole	127.87 (100-163.52) [983]
cariprazine	2.15 (1.07-4.33) [8]
brexpiprazole	3.18 (2.23-4.52) [32]

Gambling	
**Substance**	**ROR**
aripiprazole	100.66 (86.22-117.51) [1964]
iloperidone	2.16 (1.02-4.56) [7]
cariprazine	1.99 (1.19-3.31) [15]
brexpiprazole	3.49 (2.75-4.43) [71]

Hypersexuality	
**Substance**	**ROR**
lurasidone	1.81 (1.33-2.47) [42]
zuclopenthixol	2.46 (1.36-4.47) [11]
aripiprazole	18 (16.05-20.17) [899]
cariprazine	2.11 (1.13-3.94) [10]
brexpiprazole	1.6 (1.04-2.47) [21]

Hyperphagia	
**Substance**	**ROR**
ziprasidone	2.24 (1.71-2.91) [58]
lurasidone	2.74 (2.06-3.64) [50]
olanzapine	3.34 (2.9-3.83) [266]
quetiapine	1.92 (1.68-2.19) [296]
amisulpride	1.86 (1.05-3.29) [12]
risperidone	1.43 (1.21-1.69) [163]
aripiprazole	3.48 (3.05-3.98) [306]
cariprazine	2.64 (1.41-4.94) [10]
brexpiprazole	7.54 (5.94-9.56) [74]

Table S2. Cariprazine cases. The drug may have been stopped after the onset of the event (T), and Dechallenge (D) or Rechallenge (R) may have verified.

Case	age	sex	country	time to onset (dd)	stopped	other explanatory drugs	ICD
**1**	57	Male	DE	55	T (D)		hypersexuality
**2**	31	Male	US	15	T (D)		compulsive shopping
**3**		Female	US			aripiprazole	hyperphagia
**4**		Female	US				gambling
**5**		Female	US	2			hyperphagia
**6**		Female	US				food craving
**7**	30	Female	US		T	aripiprazole; brexpiprazole	compulsive sexual behaviour; compulsive shopping; gambling disorder
**8**		Female	US	1	T		hyperphagia
**9**	40	Male	US				libido increased
**10**	56	Female	US			aripiprazole	gambling disorder
**11**	47	Female	US		T (D)		hyperphagia
**12**	29	Female	US			aripiprazole	binge eating; compulsive shopping
**13**	51	Female	US		T (D)		gambling disorder
**14**	47	Female	US		T (D;R)		hyperphagia
**15**		Female	US				gambling disorder
**16**		Female	US			aripiprazole	binge eating; compulsive shopping
**17**		Male	US				gambling disorder
**18**		Male	US			aripiprazole	gambling
**19**			US				libido increased
**20**	26	Female	US				gambling disorder
**21**		Male	US		T (D)		gambling disorder
**22**		Male	DE	55	T (D)		hypersexuality
**23**	57	Male	DE		T (D)		hypersexuality
**24**		Male	DE	55	T (D)		hypersexuality
**25**		Female	US			aripiprazole; brexpiprazole	compulsive sexual behaviour; compulsive shopping; gambling disorder
**26**		Female	US			aripiprazole	compulsive sexual behaviour; compulsive shopping; gambling disorder
**27**	52	Female	US			aripiprazole	compulsive shopping; gambling disorder
**28**	55	Female	US	1		brexpiprazole	gambling; hyperphagia
**29**	22	Male	US				gambling disorder
**30**		Female	US			aripiprazole	binge eating; compulsive shopping
**31**		Female	US				gambling disorder
**32**		Female	US				hypersexuality

SESSION – Chi-Squared Statistics

Chi-squared statistics for sex distribution. 2x2 chi-squared tests for independence with Yates correction, comparing reports of men and women treated with TGAs with and without the ICD conditions. In the central cells, data are thus reported: observed (expected) [standardized residuals]. The p-value is shown before the Holm-Bonferroni correction (see Table 1).

	**TGA_any ICD**	**Other TGA**	***Marginal Row Totals***
**Female**	1274 (1430.98) [-6.65]	41447 (41290.02) [6.65]	42721
**Male**	1122 (965.02) [6.65]	27668 (27844.98) [-6.65]	28790
Marginal Column Totals	2396	69115	71511

The chi-square statistic is 43.95798. The *p*-value is <0.000001.

	**TGA_Gambling**	**Other TGA**	***Marginal Row Totals***
**Female**	902 (1049.59) [-7.27]	41447 (41299.41) [7.27]	42349
**Male**	855 (707.41) [7.27]	27668 (27835.59) [-7.26]	28523
Marginal Column Totals	1757	69115	70872

The chi-square statistic is 52.49489. The *p*-value is <0.000001.

	**TGA_Hypersexuality**	**Other TGA**	***Marginal Row Totals***
**Female**	390 (493.36) [-7.38]	41447 (41343.64) [7.38]	41837
**Male**	435 (331.64) [7.38]	27668 (27791.36) [-7.38]	28103
Marginal Column Totals	825	69115	69940

The chi-square statistic is 53.9853. The *p*-value is <0.000001.

	**TGA_Shopping**	**Other TGA**	***Marginal Row Totals***
**Female**	557 (551.62) [0.36]	41447 (41343.64) [-0.36]	42004
**Male**	363 (368.38) [-0.36]	27668 (27791.36) [0.36]	28031
Marginal Column Totals	920	69115	70035

The chi-square statistic is 0.1092905. The *p*-value is 0.740953.

	TGA_Hyperphagia	Other TGA	*Marginal Row Totals*
**Female**	229 (205.75) [2.56]	41447 (41470.25) [-2.56]	41676
**Male**	114 (137.25) [-2.56]	27668 (27664.75) [2.56]	27782
Marginal Column Totals	343	69115	69458

The chi-square statistic is 6.31942. The *p*-value is 0.011942.
